# Heat Transfer Scale Effect Analysis and Parameter Measurement of an Electrothermal Microgripper

**DOI:** 10.3390/mi12030309

**Published:** 2021-03-15

**Authors:** Lin Lin, Hao Wu, Liwei Xue, Hao Shen, Haibo Huang, Liguo Chen

**Affiliations:** 1 Robotics and Microsystems Center, Soochow University, Soochow 215006, China; 20134029004@stu.suda.edu.cn (L.L.); wuhao199110@outlook.com (H.W.); xueliwei1992@outlook.com (L.X.); 20194029003@stu.suda.edu.cn (H.S.); 2 Department of Mechanical Engineering, Xiamen Ocean Vocational College, Xiamen 361102, China

**Keywords:** electrothermal microgripper, microscale, heat effect, temperature field, parameter measurement

## Abstract

An electrothermal microgripper is an important actuator in microelectromechanical and micro-operating systems, and its temperature field analysis is the core problem in research and design. Because of the small size of an electrothermal microgripper, its microscale heat transfer characteristics are different from those of the macrostate. At present, only a few studies on the heat transfer scale effect in electrothermal microgrippers have been conducted, and the heat transfer analysis method under the macrostate is often used directly. The temperature field analysed and simulated is different from the actual situation. In the present study, the heat transfer mechanism of an electrothermal microgripper in the microscale was analysed. The temperature field of a series of microscale heating devices was measured using microthermal imaging equipment, and the heat transfer parameters of the microscale were fitted. Results show that the natural convective heat transfer coefficient of air on the microscale can reach 60–300 times that on the macroscale, which is an important heat transfer mode affecting the temperature field distribution of the electrothermal microgripper. Combined with the finite element simulation software, the temperature field of the electrothermal microgripper could be accurately simulated using the experimental microscale heat transfer parameters measured. This study provides an important theoretical basis and data support for the optimal design of the temperature controller of the electrothermal microgripper.

## 1. Introduction

As a typical actuator of a microelectromechanical system (MEMS), a microgripper is mainly used to perform operations such as gripping, moving and assembling several small objects, and it plays a vital role in the implementation of the micro-operation process [1,2,3,4,5]. Microgrippers are mainly classified as piezoelectric, electromagnetic, pneumatic, electrostatic and electrothermal according to the drive mode. Among these types, the electrothermal microgripper has attracted considerable attention owing to its advantages such as simple structure, small volume, fast response, positive driving force, strong anti-interference capability and easy control [6,7].

The electrothermal microgripper is usually made of silicon, SU-8 glue and nickel–iron alloy through the integrated circuit manufacturing process. Its feature size is generally at the micron scale, and the maximum temperature after being energised and heated generally ranges from 100 °C to 800 °C. Studies have shown that, when the size of a microelectronic device is fine to a certain extent, its heat transfer law is different from that at the conventional scale; in other words, the heat transfer scale effect occurs [8,9,10]. However, only a few studies on the heat transfer scale effect in electrothermal microgrippers have been conducted.

In a previous design and analysis of microscale heat transfer, Tuckerman et al. [11] first proposed that the significant difference between the flow and heat transfer phenomenon in micropipes and that in conventional pipes. Peirs et al. [12] reported that the air natural convective heat transfer coefficient at a scale smaller than 100 µm may reach 100 $W\cdot m^{-2}\cdot K^{-1}$, which is 10–20 times that in the macrostate. However, Nath and Chopra [13] determined that, from 6,000 Å to 400 Å, the heat conductivity of copper decreases by five times. These studies propose that the heat transfer characteristics do have a scale effect and no consensus has been reached on the heat transfer parameters.

In this study, a silicon-based electrothermal parallel-beam microgripper with a simple structure was investigated. Firstly, the heat transfer mechanism was analysed theoretically, the heat flow model was established and the fitting equation for the heat transfer parameters was obtained. Secondly, the temperature field at the micron scale was measured and characterised, and the influencing factors of the heat transfer parameters were analysed. Finally, the matching degree between the heat transfer parameters and the actual heat transfer characteristics of the electrothermal microgripper was verified by experimental comparison.

## 2. Analysis of the Heat Transfer Mechanism and Modelling

### 2.1. Analysis of the Heat Transfer Mechanism

After the electrothermal drive of the electrothermal microgripper and the application of a voltage, the current flows through the narrow hot arm and the wide cold arm. High Joule heat, temperature and thermal expansion are generated in the hot arm to drive the gripping fingers at the ends to generate a gripping action [14,15]. The heat transfer process of the electrothermal microgripper is composed of heat conduction, heat radiation and air natural convective heat transfer. These heat transfer processes at the microscale are analysed.

Previous studies of the heat transfer of electrothermal microgrippers made of silicon materials mainly focused on heat conduction, neglecting the influence of heat convection and heat radiation. The heat-carrying particles in silicon materials are phonons. Chen [16] indicated that the mean free path of phonons in semiconductors and other solids at room temperature is approximately 10–100 nm. Phonon transport is ballistic rather than diffusive only when the mean free path is larger than the characteristic scale of the device; thus, Fourier’s law based on diffusive transport does not apply. The feature size of the actuating arm of the electrothermal microgripper is the width of the hot beam and the width of the minimum clearance, i.e., generally 10 µm. Thus, the feature size of the electrothermal microgripper is larger than its mean free path, i.e., Fourier’s law in the macroscopic state is still applicable.

The radiative heat dissipation problem mainly involves the radiative heat dissipation after reaching the steady state. There is a gap between the cold arm and the hot arm of the electrothermal microgripper. The radiation scattering problem of the gap approximates the radiative transfer between plates with an equal surface area. The effect of the gap on the heat dissipation of the electrothermal microgripper should also be considered. Tien and Chen [17] divided the characteristic space into three spatial microscale fields according to the relationship between the characteristic space scale L and the phonon wavelength λc, coherence length lc and average free path λ. For silicon materials, both the coherence length and wavelength are at the nanoscale, whereas the feature size of the electrothermal microgripper is at the micron scale and larger than the coherence length and wavelength. Therefore, the continuous medium hypothesis is still applicable to heat radiation, as is the classical radiation transport model Stefan-Boltzmann law [18].

The convective heat transfer of the electrothermal microgripper occurs in the natural convective boundary layer of air. Therefore, the theories applicable to forced convection previously investigated by scholars cannot be applied directly. The convective heat transfer of the electrothermal microgripper is also an important parameter affected by the microscale effect in this study. Natural convection is a buoyancy-induced motion caused by a volume force acting on a density gradient. Therefore, when describing the differential equation for the free convection boundary layer, density, which is a physical property parameter, cannot be defined as a constant. Instead, density is a variable and serves as a function of temperature.

Then, the local natural convective heat transfer coefficient $\alpha_{x}$ and the Nusselt number ${\mathrm{Nu}}_{x}$ are derived as follows:

(1) $$\alpha_{x}=\frac{q_{w}}{t_{w}-t_{\infty }}$$

(2) Nux=αxxk=− θ ’(0)(Grx4)14=gPr(Grx4)14

where $q_{w}$ is the surface heat flow, $t_{w}$ is the surface temperature and $t_{\infty }$ is the ideal fluid temperature outside the boundary layer.

Finally, the differential exact solution is derived as follows:

(3) Nux=0.75[2Pr2Grx5(1+2Pr12+2Pr)]14

where ${\mathrm{Gr}}_{x}=\beta g\Delta {\mathrm{tx}}^{3}/v$ is the Grashof number (Gr), β is the volume expansion coefficient and Pr=v/a is the Prandtl number (where a is the thermal diffusion system).

Formula 1 indicates that Newton’s law of cooling is still valid. The convective heat transfer coefficient $\alpha_{x}$ of the actuating arm at different temperatures can be calculated by substituting the thickness, length and width of the actuating arm into Formulas (2) and (3). These formulas indicate an exponential relationship between the convective heat transfer coefficient and the range of the feature size scale, which has a significant influence on the heat transfer characteristics of the electrothermal microgripper.

### 2.2. Heat Flow Model of a Micro-Unit

On the basis of the analysis of the heat transfer mechanism, the heat transfer characteristics and macroscale of the electrothermal microgripper are quite different in the micrometre scale of the general characteristic scale. To measure the thermal parameters of heat transfer of electrothermal microgrippers, the most simplified model is used. One end of this model is connected to a thermostatic heat source matrix with a temperature of $T_{j}$, and the other end is a suspended long straight cantilever beam structure. Because the length of the hot arm is greater than its width and height, the temperature difference on the cross section can be disregarded. A micro-unit formed by two cross sections that are perpendicular to the length and dx apart on the hot arm X from the bottom is analysed.

The energy exchange on the micro-unit includes the heat conducted into the micro-unit on the left $Q_{\mathrm{cr}}$, the heat conducted from the micro-unit on the right $Q_{\mathrm{cc}}$, the heat lost by heat radiation from the sidewalls and upper and lower surfaces of the micro-unit $Q_{f}$ and the heat lost through convective heat transfer amongst the sidewalls and upper and lower surfaces of the micro-unit $Q_{d}$.

On the basis of the analysis of the microscale heat transfer mechanism presented in the previous section, the heat transfer scale effect occurs at scales smaller than 10–100 nm, which are quite different from 10 µm, i.e., the feature size of this structure. Hence, the macro Fourier formula and the heat conductivity coefficient can still be used for analysis. Therefore, the following can be obtained:

The heat conducted into the micro-unit:

(4) $$Q_{\mathrm{cr}}=-\mathrm{kwh}{\left(\frac{\mathrm{dT}}{\mathrm{dx}}\right)}_{x}$$

where k is the coefficient of heat conductivity.

The heat conducted from the micro-unit:

(5) $$Q_{\mathrm{cc}}=-\mathrm{kwh}{\left(\frac{\mathrm{dT}}{\mathrm{dx}}\right)}_{x+\mathrm{dx}}$$

The previously presented derivation shows that the Stefan–Boltzmann equation is still applicable at the microscale.

The heat lost by heat radiation from a surface:

(6) $$Q_{f}=2(w+{h)\varepsilon \sigma }_{b}(T^{4}-T_{0}^{4})\mathrm{dx}$$

where ε is the blackness and $\sigma_{b}$ is the Stefan–Boltzmann constant.

However, when the operating temperature of the electrothermal microgripper is 100 °C to 800 °C, according to Wien’s displacement law:

(7) $$\lambda_{m}T=b$$

where $b=2.898\times 10^{-3}m\cdot K$.

The main radiation wavelength is 7.77–2.7 µm, which is equivalent to the feature size of a long straight beam. Therefore, whether thermal radiation is affected by the scale effect needs to be considered. The surface emissivity of silicon in the macrostate cannot be directly applied to the microscale, and the radiation quantity of an object at the microscale can break through the Planck blackbody radiation limit, which needs to be measured experimentally.

At the microscale, Newton’s formula is still applicable. However, the convective heat transfer coefficient is quite different from that in the macro conventional-scale state, and the macroparameters cannot be directly used.

The heat lost by convective heat transfer from a surface:

(8) $$Q_{d}=2(w+h)\alpha \left(T-T_{0}\right)\mathrm{dx}$$

where α is the air natural convective heat transfer coefficient.

In the steady state, the temperature of the micro-unit remains unchanged according to the law of conservation of energy:

(9) $$Q_{\mathrm{cr}}-Q_{\mathrm{cc}}=Q_{f}+Q_{d}$$

The one-dimensional steady-state differential equation is obtained as follows:

(10) $$\mathrm{kwh}(\frac{d^{2}T}{{\mathrm{dx}}^{2}})=2\left(w+h\right)\left[{\varepsilon \sigma }_{b}(T^{4}-T_{0}^{4})+\alpha (T-T_{0})\right]\mathrm{dx}$$

In the experiment on heat transfer parameter determination, Formula 10 can be used for parameter fitting to obtain the values of blackness ε and convective heat transfer coefficient α.

## 3. Experiment and Simulation of Heat Transfer Parameter Measurement

### 3.1. Experimental Setup

**Figure 1:**
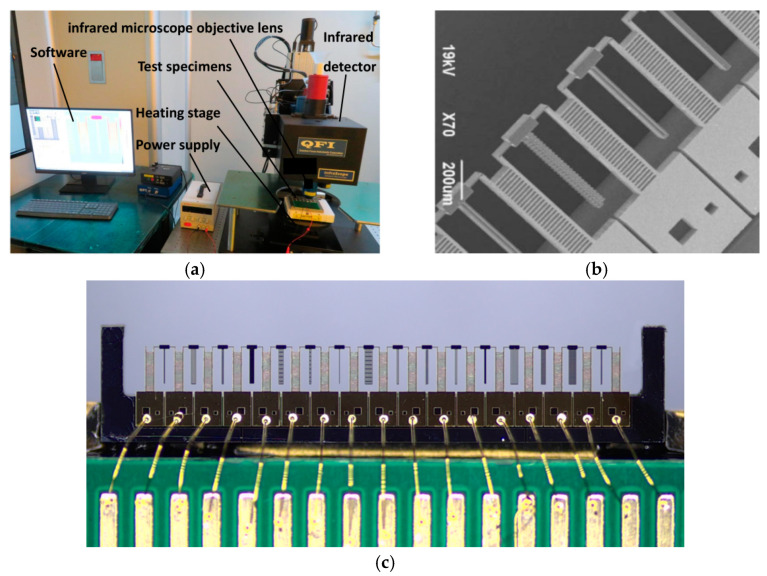
Experimental setup and test specimens: (**a**) Experimental System of micro-infrared thermometry; (**b**) electron micrograph of test specimens; (**c**) microscopic imaging of test specimens.

Because of the lack of research on the heat transfer parameters of silicon-based electrothermal devices in the micron scale, there is no reliable heat transfer simulation and analysis tool in this direction. The commonly used thermocouple contact temperature measurement method easily affects the distribution of the temperature field. In the experiment, the micro-infrared temperature measurement technology is used to measure the temperature distribution in the entire field of view (FOV), which can comprehensively reflect the temperature distribution of the device, and the numerical measurement and simulation analysis of heat transfer parameters are conducted. The microthermal imaging measurement system is shown in Figure 1a, which is mainly composed of a micro-infrared imager, display and power supply.

The model of the micro-infrared imager is the QFI TMS infrared analyser made in USA, which consists of an infrared detector, infrared microscope objective lens, image acquisition card, software processing system and stage. The magnification (and the FOV) of the equipment is 1X (9 mm × 9 mm FOV), 5X (2.46 mm × 2.46 mm FOV) and 15X (0.82 mm × 0.82 mm FOV). The minimum resolution is 2.7 µm, the temperature sensitivity is 0.1 °C, the infrared band is mid-infrared (3–5 µm) and the temperature range of the heating stage is 20 °C to 200 °C. The imager has different material emissivity calibration algorithms, which can eliminate the influence of the different surface emissivities of silicon and gold on the upper surface of the test specimens, reflect the real temperature of the test specimens and meet the experimental requirements.

In the experiment, a variety of cantilever beam test specimens, such as solid long straight beam and microgap, are made. To improve the measuring efficiency, a multiple cantilever beam structure is integrated into a part of the body, as shown in Figure 1b.

For this part, the thickness of the structure layer is 50 µm, the thickness of the substrate layer is 350 µm and a 200 nm gold pad is sputtered on the surface, as shown in Figure 1c. The feature size of conventional electrothermal microgrippers and other electrothermal MEMS devices is generally 3–25 µm. The main feature size of the test specimens is 10 µm in width, which is limited by the processing technology and yield. The manufacturing technology of the test specimens is the same as that of the electrothermal microgripper.

**Figure 2:**
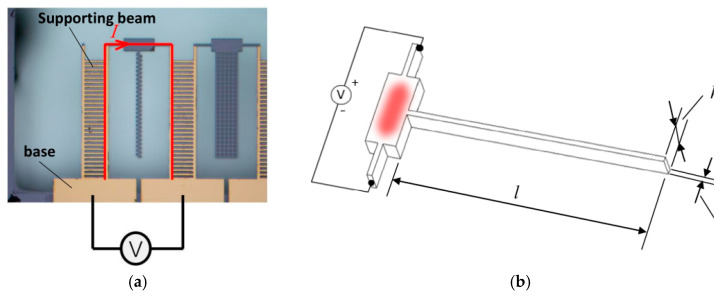
Working principle of the experiment: (**a**) Diagram of loading voltage; (**b**) schematic diagram of device heating.

To eliminate the influence of Joule heat, matrix heat transfer and other factors and amplify the effect of convection and radiation, the cantilever beam is designed as shown in Figure 2a. The positive and negative electrodes are connected to the lead pads at both ends of the test specimens. After the direct-current-regulated power supply is connected, the current flows through the supporting beams and cuboid at the top. Joule heat can be generated in all current flow areas and affected by the heat conduction of the base and supporting beams. The highest temperature is observed at the top rectangular area, which can become a constant-temperature heat source, as shown in Figure 2b. All kinds of cantilever test specimens are connected to the cuboid at the top and suspended at the other end, which can be regarded as a heat balance body that absorbs heat from a constant-temperature heat source and dissipates heat through heat convection and radiation.

### 3.2. Parameter Measurement and Fitting

Of the test specimens, the long straight beam with the simplest structure is measured. The dimensions of the long straight beam are l = 50 µm, w = 10 µm and h = 50 µm.

The electrothermal microgripper is made of monocrystalline silicon, of which the characteristic parameters at the macro conventional-scale state are listed in Table 1 [19].

**Figure 3:**
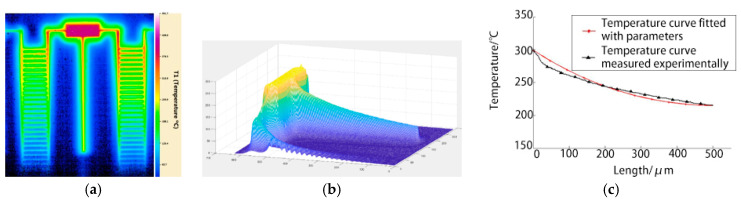
Fitting curve of heat transfer parameters: **(a**) Temperature field of long straight beam; (**b**) numerical Matrix at 300 °C; (**c**) fitting curve of the heat transfer parameters.

The temperature field distribution measured using the microscopic thermal imaging device is shown in Figure 3a. According to the requirements of the infrared temperature measurement system, the temperature of the experimental table should be constant at 45 °C. The temperature of the cuboid constant-temperature heat source is 300 °C. The terminal temperature decreases to 225 °C after heat dissipation by convection and radiation on the beam. The experimental images are converted into a numerical matrix for linear transformation processing, as shown in Figure 3b.

For the long straight beam, Formula 10 is used for parameter fitting, obtaining the blackness ε=0.8 and the convective heat transfer coefficient α=1558 W/(m^2^·°C). The fitting curve is shown in Figure 3c.

The heat radiation coefficient obtained by fitting basically coincides with that in the macro state. The convective heat transfer coefficient is 60–300 times that in the macrostate, as shown in Table 1, which is also consistent with the relationship between convective heat transfer coefficient and feature size in the analysis of the heat transfer mechanism.

### 3.3. Influence of the Heat Transfer Parameters on the Temperature Fields

**Figure 4:**
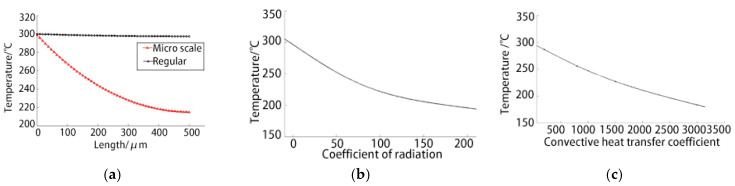
Influence of the heat transfer parameters on the temperature field: (**a**) Comparison of the experimental results; (**b**) effect of the radiation coefficient; (**c**) effect of the convective heat transfer coefficient.

The differential equation for the one-dimensional temperature distribution on the long straight beam is used to analyse the influence of different heat transfer parameters on the temperature fields. As shown in Figure 4a, the black curve denotes the temperature distribution when the heat transfer macroparameters are used. Its air natural convective heat transfer coefficient is set to 10 W/(m^2^·°C) in the macrostate. The blackbody coefficient is set to 0.9. The red curve denotes the fitting curve of the experiment in this study. Notably, there is a significant difference between the two curves. The terminal temperature of the long straight beam decreases by only 0.5 °C when the heat transfer macroparameters are used. Macroscale heat transfer analysis is far from being used for heat transfer analysis at the 10 µm scale.

Figure 4b shows the changes of the terminal temperature with the heat radiation coefficient. According to the analysis, the radiation effect at the micron scale will not be significantly enhanced. As shown in the figure, even if the heat radiation coefficient increases to 10, the heat transfer effect does not affect the temperature field.

As shown in Figure 4c, there is a linear relationship between the terminal temperature of the long straight beam and the convective heat transfer coefficient. The convective heat transfer coefficient also has a significant effect on the temperature field.

### 3.4. Simulation Modelling

The temperature distribution of the micro-actuator can be solved according to the differential equation and boundary condition. However, in engineering applications, the analytical or numerical solution of differential equations for heat transfer can only be applied to a few simple geometries. For the analysis, design and optimisation of most practical complex structures, the simulation is still a useful method.

**Figure 5:**
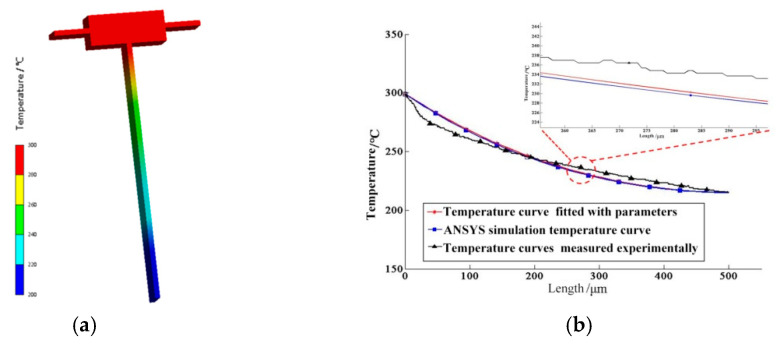
Simulation model and comparison: (**a**) Finite element simulation model; (**b**) comparison of the temperature curves.

Because the boundary condition is closed at the temperature of the cuboid, the influence of Joule heat, heat transfer of the base and heat radiation and convection of the supporting arm can be disregarded during the simulation. The ambient temperature is set to 45 °C. The boundary condition is added. The temperature of the cuboid is 300 °C. All surfaces are selected to add heat radiation and convective heat transfer. The convective heat transfer coefficient is set to 1558 W/(m^2^·°C). The simulation results are shown in Figure 5a.

The comparison of the actual temperature distribution on the long straight beam, parameter fitting curve and simulation results is shown in Figure 5b.

The curve of the temperature distribution on the simulation beam is completely consistent with the parameter fitting curve, with a maximum error of 0.8 °C.

## 4. Determination of the Influencing Factors of the Heat Transfer Parameters

Because the heat of the test specimens is mainly dissipated to the outer space and the gap between the parallel beams by convection and radiation, the thermal radiation coefficient is less affected by the size effect. The influence of thermal radiation on the temperature field distribution can also be disregarded. Thus, this research only focuses on the influencing factors of the convective heat transfer coefficient.

### 4.1. Effect of Feature Size

**Figure 6:**
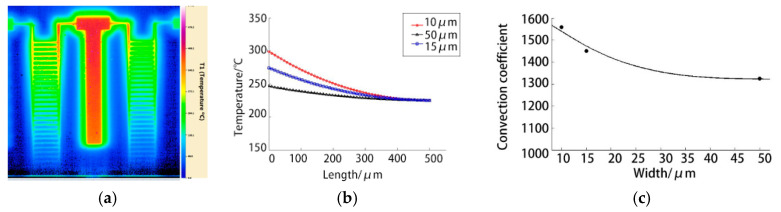
Effect of feature size on the heat transfer parameters: (**a**) Measured temperature field of cantilever beam test specimens with a width of 50 µm; (**b**) fitting results of the heat transfer parameters; (**c**) variation of the heat transfer parameters with characteristic size.

To analyse the effect of different feature sizes of electrothermal microgrippers on the heat transfer parameters, two cantilever beam devices with widths of 15 and 50 µm. Moreover, the same length and thickness were designed for contrastive analysis. The measured temperature field of the cantilever device with a width of 50 µm is shown in Figure 6a. After recording the measured temperature fields in different sizes, the convective heat transfer coefficient is adjusted. The fitting results of the measured temperature field curves obtained are shown in Figure 5b. The convective heat transfer coefficients on the surface of the 50, 15 and 10 µm devices obtained are 1350, 1425 and 1558 W/(m^2^·°C), respectively. The variation trend of the surface convective heat transfer coefficient with the device size obtained by further fitting is shown in Figure 6c.

The experimental results indicate that the convective heat transfer coefficient gradually increases with the decrease in the device size, which represents a gradual increase in the capability to dissipate heat. Natural convective heat transfer mainly caused by density difference is characterised by the size of the Grashchev number Gr (containing buoyant force). The smaller the feature size of the solid beam is, the smaller the volume of the air around it, the larger the air temperature gradient, the higher the buoyant force and the more the convective heat transfer.

### 4.2. Effect of Microgap

**Figure 7:**
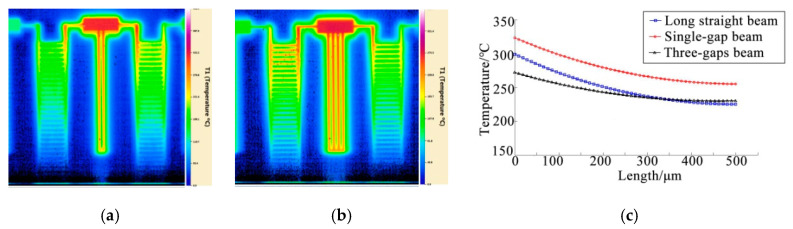
Measured temperature field and fitting curve of the gap structures: (**a**) Temperature field of a single-gap structure; (**b**) temperature field of a three-gap structure; (**c**) fitting curve of the gap heat transfer parameters.

A microgap is a common structure in an electrothermal microgripper. It may appear between the cold and hot arms of the electrothermal parallel beam and actuating arms of the V-shaped gripper. To analyse the heat dissipation between gaps at the microscale, two specimens with single-gap and three-gap structures are designed. The width of both gap and beam is 10 µm, and the length and thickness remain unchanged. The measured temperature fields are shown in Figure 7a,b.

The convective heat transfer coefficient in the gap is changed (with other parameters unchanged) to fit the temperature field. The temperature field curves obtained from fitting are shown in Figure 7c. In Figure 7c, the convective heat transfer coefficients between the gaps of single-gap and three-gap specimens are 625 and 328 W/(m^2^·°C), respectively, both of which are far less than those of the solid long straight beam. The results also indicate that the heat dissipation capacity of the cantilever beam with a gap structure is weakened.

Compared with the air near the outer surface of the structure, the temperature gradient of the air between the gaps in the horizontal direction is significantly reduced, the buoyant force of air is weakened. Moreover, both walls of the gaps exhibit viscous resistance to the airflow; thus, the natural convective heat transfer is further reduced. Compared with the single-gap structure, the three-gap structure is a larger heat source, the volume of the surrounding air is larger, the temperature gradient of the air is smaller, the buoyant force is weaker and the convective heat transfer capability is weaker.

## 5. Experimental Verification

To verify whether the heat transfer parameters measured in the experiment match the actual heat transfer law of the electrothermal microgripper, the measured heat transfer parameters and the heat transfer macroparameters are substituted into the electrothermal parallel-beam microgripper, and the temperature distribution obtained is compared with the measured temperature field.

**Figure 8:**
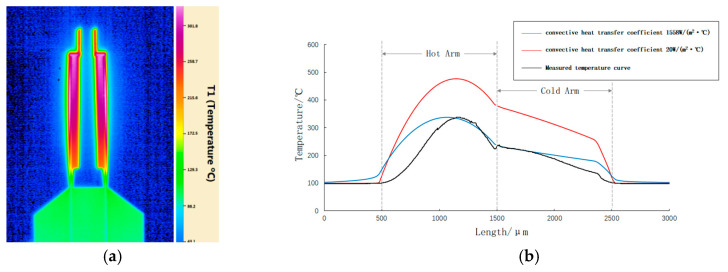
Comparison of the heat transfer parameters: (**a**) Measured temperature field of the microgripper; (**b**) comparison of the temperature curves.

Figure 8a shows the measured temperature field distribution of the electrothermal parallel-beam microgripper when the voltage is 6.18 V. The maximum temperature is 344 °C, which occurs in the middle part of the hot arm. Figure 8b shows the temperature field distribution curves for the measured and convective heat transfer macroparameters and the experimentally measured convective heat transfer parameters.

Figure 8b shows that the blue curve (experimentally measured parameters) is highly consistent with the black curve (measured parameters). Meanwhile, the maximum temperature of the red curve (macroparameters) is as high as 477 °C, which deviates from the measured temperature curve. The comparison results show that the heat transfer parameters at the microscale measured herein are consistent with the actual conditions. Meanwhile, the effect of the microscale on the heat transfer characteristics of electrothermal microgrippers is verified.

## 6. Conclusions

In this study, the heat transfer mechanism of an electrothermal microgripper in the microscale is theoretically analysed, a heat flow model is established and a fitting equation for the heat transfer parameters is derived. Thereafter, the microscale heat transfer parameter measurement test specimens are made, and the temperature field measurement, parameter fitting and finite element simulation comparison of different microscale test specimens are conducted. Simultaneously, the characteristic size is analysed. Finally, through the comparison of the experimental results, the measured parameters are determined to be consistent with the actual heat transfer law of the electrothermal microgripper in which the convective heat transfer coefficient has a significant effect on the temperature field, whereas the thermal radiation coefficient has a negligible effect on the temperature field. Moreover, the convective heat transfer coefficient in the microscale can reach 60–300 times that in the macrostate, which is the most influential factor. According to the important parameters of microscale heat transfer, the heat transfer macroparameters cannot be directly used to analyse the heat transfer characteristics of electrothermal microgrippers.

The results show that, with the decrease in the size of the driving arm of the electrothermal microgripper, the behaviour of the thermal fluid in the structure will seriously deviate from the law described by the traditional heat transfer theory and show a strong size effect. This will provide an important theoretical basis and data support for the optimal design of the temperature controller and expansion of the application field of the electrothermal microgripper.

## Figures and Tables

**Table 1:** Parameters of silicon at the conventional scale.

Heat Transfer Parameter	Numerical Value
Coefficient of heat conductivity k (W/(m·°C))	148
Blackness ε	0.6
Convective heat transfer coefficient α (W/(m^2^·°C))	5–25
Stefan–Boltzmann σ_b_ (W/(m^2^·K^4^))	5.67 × 10^−8^

## Data Availability

The data presented in this study are available on request from the corresponding author. The data are not publicly available due to privacy restrictions.
